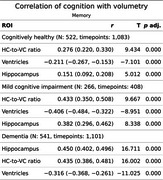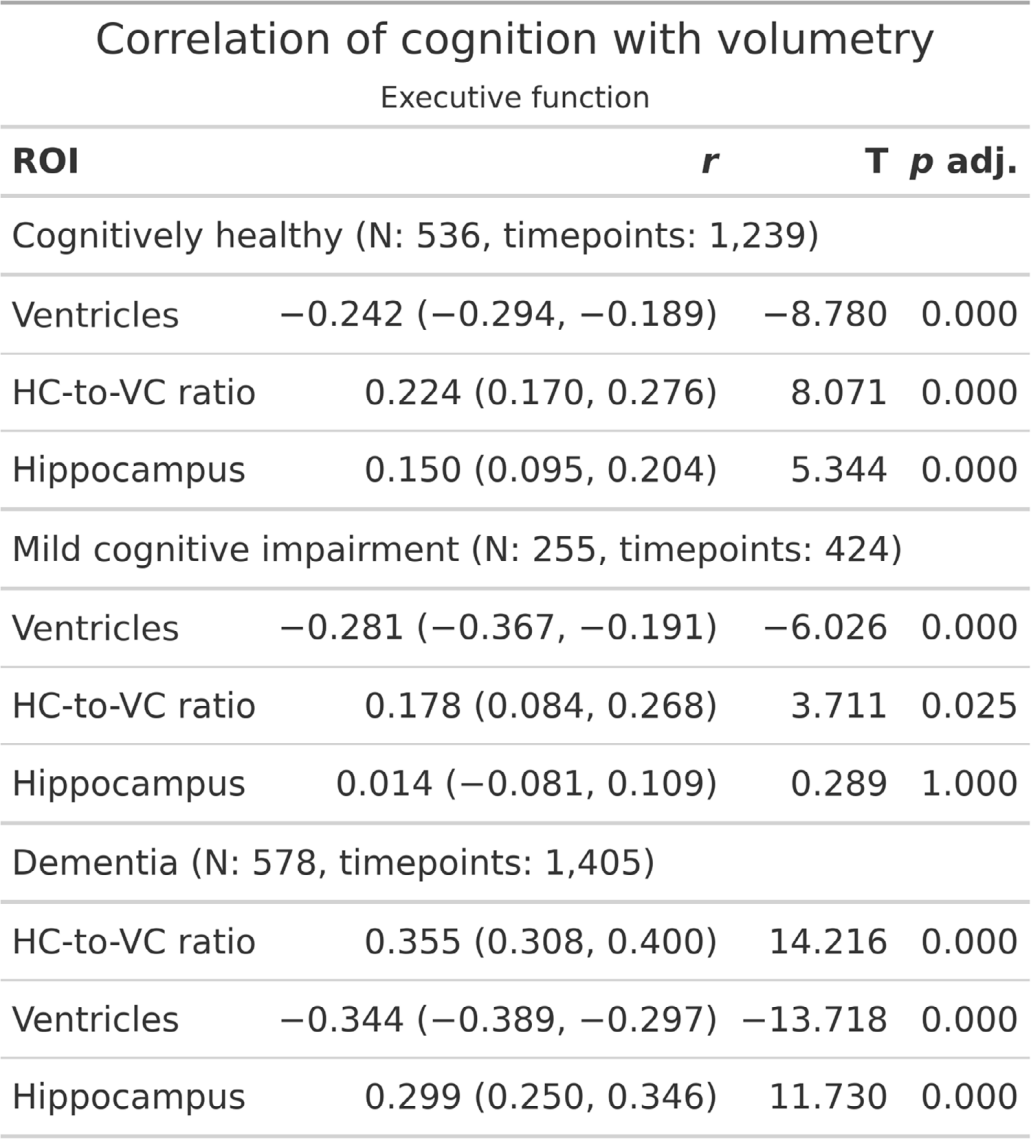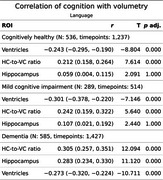# The Role of Negative Space: Lateral Ventricular Expansion Is a Better Correlate of Cognition Than Hippocampal Volume

**DOI:** 10.1002/alz70856_106003

**Published:** 2026-01-08

**Authors:** Sofia Fernandez‐Lozano, D Louis Collins, Vladimir S Fonov, Alzheimer's Disease Neuroimaging Initiative (ADNI)

**Affiliations:** ^1^ Department of Neurology and Neurosurgery, McGill University, Montreal, QC, Canada; ^2^ McConnell Brain Imaging Centre, Montreal Neurological Institute, McGill University, Montreal, QC, Canada; ^3^ Department of Biomedical Engineering, McGill University, Montreal, QC, Canada; ^4^ Laboratory of Neuro Imaging (LONI), University of Southern California, Los Angeles, CA, USA

## Abstract

**Background:**

The hippocampus (HC) is a key biomarker in Alzheimer's disease (AD), yet the relationship between ventricular enlargement—a marker of brain atrophy—and cognition remains underexplored. We evaluate whether lateral‐temporal horn ventricular measurements are a viable correlate for cognition.

**Method:**

From the ADNI dataset, we analyzed cognitive data from 481 cognitively healthy (CH), 548 mild cognitively‐impaired (MCI), and 222 AD individuals. We obtained factors for memory, language, and executive function from validated confirmatory factor analysis.

We used MRI data to segment the HC and the temporal horns of the lateral ventricles (LV) with a Convolutional Neural Network. Volumes were adjusted for intracranial volume (ICV). We calculated the HC‐to‐Ventricle ratio (HVR), [HC / (HC + LV)], a medial‐temporal integrity measurement.

Finally, with Pearson correlations, we assessed the relationships between cognition and HC, LV, and HVR.

**Result:**

LV showed stronger negative correlations with cognition than HC, particularly in CH and MCI.

Memory: In MCI, HVR (*r* = .43) and LV (*r* = ‐0.40) correlated more strongly with memory than HC (*r* = .38). In AD, HC (*r* = .45) and HVR, (*r* = .44) outperformed LV (*r* = .32).

Executive function: Across groups, LV (CH, r = ‐.24; MCI, r = ‐.28; AD, r = ‐.34) and HVR (CH, *r* =.22, MCI, *r* = .18, AD, *r* = .36) exceeded HC (CH, *r* = .15, AD, *r* = .3).

Language: In CH and MCI, only LV and HVR, and not HC, were significantly associated with the latent score; with LV (CH, r = ‐.24; MCI, r = ‐.3) slightly outperformed HVR (CH, *r* = .21; MCI, *r* = .24). For the patients with AD, the correlation of HC (*r* = .28) while significant, was still weaker than HVR (*r* = .31).

**Conclusion:**

Lateral ventricular enlargement correlates more strongly with cognitive decline than hippocampal atrophy alone. The HVR, by design, integrates information from both hippocampal atrophy and ventricular expansion, making it a more comprehensive and robust measure of medial‐temporal integrity. These results highlight the importance of ventricular expansion as a key biomarker in AD research and clinical assessments.